# Artificial Intelligence and the Expanding Universe of Cardio-Oncology: Beyond Detection Toward Prediction and Prevention of Therapy-Related Cardiotoxicity—A Comprehensive Review

**DOI:** 10.3390/diagnostics16030488

**Published:** 2026-02-05

**Authors:** Miruna Florina Ștefan, Lucia Ștefania Magda, Dragoș Vinereanu

**Affiliations:** 1 University and Emergency Hospital of Bucharest, 050098 Bucharest, Romania; miruna-florina.stefan@drd.umfcd.ro (M.F.Ș.); vinereanu@gmail.com (D.V.); 2 Cardiology and Cardiovascular Surgery Department, University of Medicine and Pharmacy Carol Davila Bucharest, 020021 Bucharest, Romania

**Keywords:** cardio-oncology, cancer therapy–related cardiotoxicity (CTRCD), artificial intelligence (AI), machine learning (ML)

## Abstract

**Background:** Cardiotoxicity is a major limitation of chemotherapy and radiotherapy for thoracic and systemic cancers, contributing significantly to morbidity and mortality among survivors. Early prediction and prevention are critical to balance oncologic efficacy with cardiovascular safety. Artificial intelligence (AI) offers powerful tools to improve risk stratification, enable earlier detection of subclinical injury, and guide treatment planning in cardio-oncology. **Methods:** We performed a comprehensive review of the literature on AI applications for cancer therapy-related cardiotoxicity. Evidence was identified from PubMed, Scopus, and Web of Science, focusing on electrocardiography, biomarkers, proteomics, extracellular vesicles, genomics, advanced imaging (echocardiography, cardiac magnetic resonance, computed tomography, nuclear imaging), and radiotherapy dose modeling (dosiomics). Translational insights from animal models and in vitro systems were also included. Methodological quality was appraised with reference to TRIPOD-AI, PROBAST-AI, and CLAIM standards. **Results:** AI applications span multiple domains. Machine learning models integrating biomarkers, exosomes, and extracellular vesicles show promise for noninvasive early detection. Deep learning enables automated analysis of echocardiographic strain and cardiac MRI mapping, while radiomics and dosiomics approaches combine imaging with cardiac substructure dose maps to predict and prevent late radiation-induced injury. Preclinical studies demonstrate AI-driven advances in small-animal imaging, histopathology quantification, and multi-omics data integration, supporting the discovery of translational biomarkers. Despite encouraging performance, most models remain limited by small cohorts, methodological heterogeneity, and scarce external validation. **Conclusions:** AI has the potential to transform cardio-oncology by shifting from reactive detection to proactive prevention of cardiotoxicity. Future research should prioritize multimodal integration, harmonized multicenter datasets, prospective validation, and guideline-based clinical trials. As emerging data are incorporated, the field is expanding rapidly—dynamic, complex, and evolving.

## 1. Introduction

Cancer and cardiovascular diseases are among the most important causes of death worldwide. The World Cancer Research Fund International reported 18.7 million cancer cases in the year 2022, with breast and lung cancer being the most frequent [1].

Moreover, cardiovascular disease is a leading cause of death in cancer survivors, among the ~17 million cancer survivors in the United States [2]. Evidence indicates that individuals with cancer face a two- to six-fold increase in cardiovascular mortality compared with the general population. As advances in oncology continue to reduce cancer-related deaths, the importance of comprehensive cardiovascular assessment and management becomes even more pronounced. Effectively addressing cardiovascular risk is now a critical component of optimizing long-term outcomes for cancer patients and survivors [3].

Cardiovascular toxicity—often referred to as *cardiotoxicity*—is defined by the 2022 European Society of Cardiology Cardio-Oncology guidelines as injury to the heart muscle or broader cardiovascular system resulting from cancer therapies. Although treatments such as chemotherapy and radiation are integral to effective cancer management, they can inadvertently impact cardiac health. These effects span a wide spectrum, from subtle, asymptomatic changes in cardiac performance to severe and potentially life-threatening complications, including heart failure. While most cardiotoxic complications arise during or shortly after treatment, some cancer therapies can lead to cardiac events that emerge many years—even decades—after therapy has ended [4].

Conventional chemotherapies, as well as endocrine therapies, targeted or immunotherapies, and radiation therapy can all cause cardiovascular toxicities [4]. Among individuals undergoing cancer therapies—including chemotherapy, radiotherapy, and targeted agents—approximately 20% may develop some degree of myocardial dysfunction, with an estimated 7–10% progressing to cardiomyopathy or heart failure [5]. Remarkable advances in cancer therapy have significantly improved long-term survival. However, these gains are increasingly tempered by cardiovascular complications associated with treatment, which can lead to therapy interruptions, reduced tolerance of cancer regimens, and ultimately worse oncologic and cardiovascular outcomes [6]. Before starting any cancer treatment, a comprehensive baseline evaluation should be made and optimization of the treatment and pre-existing cardiovascular (CV) risk factors and/or CV disease is recommended [4]. There is a pressing need to develop non-invasive, accesible and low-cost tools for the early identification of survivors at risk for cardiovascular disease, to facilitate optimal screening, timely diagnosis, and early intervention.

**Figure 1:**
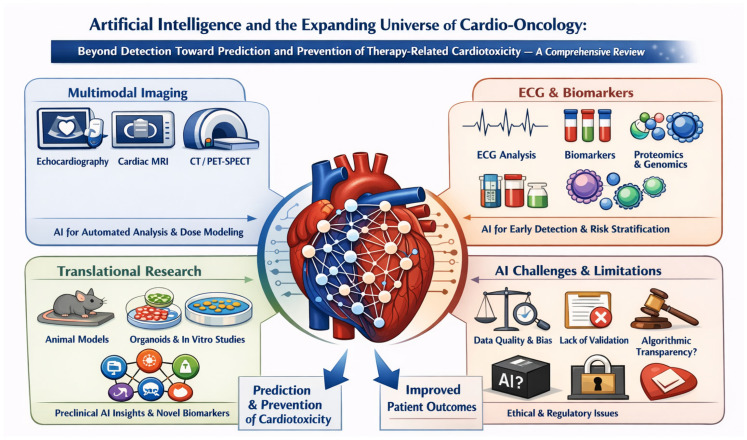
Overview of current and emerging applications of artificial intelligence in cardio-oncology for the prediction and prevention of therapy-related cardiotoxicity.

As many patients with cancer undergo frequent surveillance imaging and other diagnostic testing, a substantial amount of clinical data is generated that could be leveraged to assess an individual’s risk for cardiovascular complications of therapy. Over time, this growing volume of structured and unstructured information—spanning imaging, laboratory values, ECGs, clinical notes, genomics and proteomics—offers an underused opportunity to develop more accurate and dynamic risk prediction tools, particularly when integrated with advanced analytical methods such as machine learning and artificial intelligence (AI) (Figure 1).

Several risk-assessment and prediction tools are currently in use or under development to help identify patients at heightened risk for cardiotoxicity and guide clinical management. Incorporating the vast amount of clinical, imaging, and molecular data that can be leveraged by AI could further accelerate the development of more refined, dynamic, and easily applicable cardiotoxicity prediction scores, ultimately enhancing their utility in real-world practice. Consequently, the field of AI-assisted precision cardio-oncology is rapidly advancing toward greater personalization and precision, with a strong focus on early prevention and individualized treatment before, during, and after cancer therapy [7].

## 2. Cancer Therapy-Related Cardiotoxicity: An Overview

Anthracyclines are among the most widely used and effective chemotherapeutic agents for a broad spectrum of malignancies—including breast cancer, sarcomas, leukemias, and lymphomas—where they play a central role in achieving high response rates and improving survival. However, their clinical utility is constrained by a well-recognized risk of cardiotoxicity, which can manifest acutely during treatment or years later, potentially leading to irreversible cardiac dysfunction. Cardiac dysfunction in this setting refers to a spectrum of structural and functional changes in the heart, most notably a reduction in its ability to pump blood efficiently. This often appears as a decline in left ventricle ejection fraction (LVEF) or abnormalities in myocardial strain, changes that may progress to symptomatic heart failure if not promptly identified [8].

Another very commonly used chemotherapeutic agent, 5-fluorouracil (5-FU) and its metabolic precursor, capecitabine, are fluoropyrimidines used in various cancer treatments, with cardiotoxicity typically manifesting during the first chemotherapy cycle. This cardiotoxicity, most often presenting as chest pain due to coronary vasospasm, can also lead to more severe complications such as myocardial infarction, arrhythmias, cardiomyopathy, and sudden cardiac death. The phenomenon appears infrequent but genuine, independent of dose, and may be related to continuous infusion schedules. To date, classic cardiac risk factors do not reliably predict its occurrence. Therefore, patients receiving 5-FU should be closely monitored, and treatment should be discontinued if cardiac symptoms develop [9].

Paclitaxel and docetaxel, microtubule-targeting agents commonly used to treat breast, head and neck, and gastrointestinal cancers, have been associated with cardiotoxic effects, including abnormal cardiac conduction. Cardiotoxicity related to these taxanes occurs in approximately 2.3% to 8% of patients and may manifest as bradycardia, atrioventricular block, hypotension, arrhythmias, and ventricular dysfunction [10,11].

Cyclophosphamide-induced cardiotoxicity is a significant concern, particularly when administered at high doses, and is primarily linked to its toxic metabolite acrolein. This metabolite promotes pathological changes such as myocyte necrosis, inflammation, and cardiac hypertrophy, contributing to cardiomyopathy and heart failure [12].

Cisplatin and other platinum derivatives are widely used chemotherapeutic agents effective against multiple malignancies, but their clinical utility is limited by renal and cardiac toxicities. Cardiotoxicity manifests as electrocardiographic changes and various arrhythmias, including ventricular arrhythmias, supraventricular tachycardia, atrial fibrillation (AF), occasional sinus bradycardia, and complete atrioventricular block. Additionally, cisplatin-induced endothelial damage, inflammation, and apoptotic pathways contribute to myocardial injury, with downstream cardiac remodeling and degeneration [13].

Trastuzumab is a monoclonal antibody targeting HER2, commonly used in HER2-positive breast cancer. While effective, it can cause reversible cardiotoxicity, primarily manifesting as heart failure and decreased left ventricular function. This cardiotoxicity results from HER2 inhibition, which disrupts cardiac cell survival pathways, leading to oxidative stress and myocyte dysfunction. Unlike anthracycline-induced damage, trastuzumab-related effects are often reversible with treatment cessation and can be managed with cardiac monitoring and protective therapies [14].

Angiogenesis inhibitors can cause adverse effects such as hypertension, thrombosis, QT interval prolongation, and left ventricular dysfunction. The latter may result partly from direct cardiomyocyte toxicity worsened by hypertension [15].

Ibrutinib, a potent Bruton tyrosine kinase inhibitor for B-cell lymphomas, is associated with an increased incidence of AF, ranging from 4% to 16%, with a median onset around 2.8 months after starting therapy. New-onset AF in cancer patients elevates the risk of heart failure and thromboembolism. Management is challenging due to drug interactions with common AF therapies. Clinicians should be vigilant for AF development and consider tailored algorithms for management in patients receiving ibrutinib [16].

Proteasome inhibitors like carfilzomib have significantly advanced multiple myeloma treatment but are associated with notable cardiovascular adverse events. These include heart failure, systemic and pulmonary hypertension, arrhythmias, and acute coronary syndrome. Cardiovascular risks are heightened, with heart failure and hypertension being common, and events often occur early during treatment, typically within the first three months [17].

There is evidence that estrogen deprivation caused by aromatase inhibitors (such as anastrozole, letrozole, exemestane) and selective estrogen receptor modulators like tamoxifen, used to treat early and advanced breast cancers, may increase the risk of ischemic heart disease. Long-term use of aromatase inhibitors for four years or more has been associated with a higher risk of ischemic heart disease and arrhythmias compared to shorter exposure or no treatment. However, the overall risk of fatal cardiovascular events appears low, and findings emphasize the importance of cardiovascular risk assessment and monitoring during endocrine therapy [18,19].

Immune checkpoint inhibitors (ICI) activate T cells to enhance the immune response against cancer cells, but can cause myocarditis, a rare yet potentially fatal immune-related adverse event. ICI-associated myocarditis often occurs within weeks of treatment initiation and may present with cardiac symptoms, arrhythmias, or be asymptomatic. It involves autoimmune infiltration and inflammation of the myocardium [20].

Cardiotoxicity from radiation therapy (RT) can present as nearly all forms of cardiovascular disease, with atherosclerosis being the most common. RT causes endothelial damage, oxidative stress, inflammation, and fibrosis, promoting accelerated atherosclerosis and coronary artery disease. Other complications include pericarditis, arrhythmias, cardiomyopathies, conduction abnormalities, and heart failure. These effects may appear months to years after treatment, depending on the specific cardiac pathology, RT dose, and patient risk factors. Advanced RT techniques aim to minimize heart exposure to reduce these risks, but the results in the long term are still to be determined [21,22].

## 3. Why Artificial Intelligence in Cardio-Oncology?

AI applications in cardio-oncology span a wide range of domains and are rapidly transforming both research and clinical practice. Machine learning (ML) models are being developed to integrate diverse sources of data—including circulating biomarkers, advanced imaging modalities and radiomics, electrocardiograms (ECGs), as well as genomic, proteomic, and metabolomic profiles. By combining these multidimensional datasets, AI enables a more comprehensive understanding of cardiovascular risk and treatment response in cancer patients. In addition, preclinical studies using animal models are increasingly incorporating AI-driven analytics to uncover novel mechanisms of cardiotoxicity and to accelerate the development of preventive or therapeutic strategies.

Moreover, an important advantage of AI lies in its ability to minimize subjectivity in data interpretation. By providing consistent, automated analyses, AI reduces both intra- and inter-observer variability, leading to more objective, reproducible, and reliable results. This consistency is particularly valuable in cardio-oncology, where subtle changes in imaging, biomarkers, or physiological signals can have critical implications for early diagnosis and treatment decisions.

AI in cardio-oncology requires understanding its different forms. ML encompasses algorithms that identify patterns in data to make predictions or classifications without explicit rule-based programming. Model performance largely depends on the quality and relevance of manually selected features informed by domain expertise. Classical ML methods—such as logistic regression, random forests, support vector machines, and gradient boosting—have been extensively applied in cardiovascular and oncologic research to predict treatment-related cardiotoxicity. These models can integrate clinical, laboratory, and imaging data to estimate, for example, the risk of anthracycline-induced ventricular dysfunction or ICI–related myocarditis. ML approaches perform best with structured datasets of moderate size and remain relatively interpretable, allowing clinicians to discern key predictive factors [23]. In ML, the input dataset is typically divided into three subsets: a training set, a validation set, and a test set. The training set, usually the largest portion, is used to develop and fit the model. The validation set is then applied to monitor performance during development and to optimize the model’s parameters. Finally, the test set—composed of previously unseen data—is used to provide an unbiased evaluation of the model’s overall performance and its ability to generalize to new samples [24].

On the other hand, Deep learning (DL), a subset of ML, employs artificial neural networks with multiple interconnected layers, enabling automatic learning of complex hierarchical features—hence the term “deep.” Architectures such as convolutional, recurrent, and transformer networks can process unstructured data, including medical images, ECG waveforms, and genomic sequences, without manual feature engineering. In cardio-oncology, DL has advanced automation in echocardiographic segmentation, strain analysis, and cardiac MRI mapping, facilitating earlier detection of subclinical myocardial injury [25].

Quer et al. pointed out different approaches in using AI for the interpretation of different investigations and event prediction. Traditional rules-based algorithms rely on explicitly programmed rules to interpret data, such as computerized ECG interpretation, where the decision-making process is fully understood. In contrast, ML algorithms learn patterns from data. **Supervised learning** uses labeled examples (e.g., ECGs with known diagnoses) to automatically identify features that predict outcomes, though it is limited by the provided labels. **Unsupervised learning** finds patterns in unlabeled data, clustering examples based on inherent similarities, similar to a medical student classifying ECGs without prior instruction. **Reinforcement learning** involves iterative feedback from the environment to improve predictions over time [26]. The most frequently used techniques in ML and DL generally fall within the two primary categories of ‘supervised learning’ and ‘unsupervised learning’. One example of this approach is the unsupervised development of models that identify key patient characteristics using electronic health record data [27].

After development, an algorithm must be validated on a separate dataset that it has not seen before. Common validation metrics include: (1) **discrimination,** which measures the model’s ability to distinguish between different outcomes (typically reported as the area under the curve [AUC] or c-statistic), and (2) **calibration,** which assesses how closely the model’s predicted risks match the observed outcomes. This step is crucial to ensure the algorithm provides reliable, high-quality data applicable to real-world clinical practice [28,29].

**TRIPOD-AI, PROBAST-AI**, and **CLAIM** are emerging standards designed to improve the transparency, quality, and reliability of artificial intelligence research in healthcare. **TRIPOD-AI** extends the original TRIPOD reporting guideline to address the specific challenges of developing and validating AI-based prediction models, ensuring that studies provide enough detail for reproducibility, critical appraisal, and clinical translation [30]. **PROBAST-AI** complements this by offering a structured framework to assess the risk of bias and applicability of AI-driven prediction tools, accounting for issues such as data handling, algorithmic transparency, and validation methods [31]. Meanwhile, the **CLAIM** (Checklist for Artificial Intelligence in Medical Imaging) guideline provides standardized reporting requirements for AI studies in medical imaging, promoting rigor in data curation, model development, evaluation, and clinical integration [32]. Together, these frameworks help align AI research with robust methodological and reporting standards, facilitating safer and more trustworthy implementation of AI tools in clinical practice.

Precision medicine moves beyond a one-size-fits-all approach by tailoring healthcare to patient subgroups based on genetic, environmental, and experiential variability, aiming to optimize outcomes, reduce adverse effects, and enable personalized monitoring and interventions. Increasingly, AI is amplifying this paradigm by integrating vast and heterogeneous datasets.

## 4. Materials and Methods

### 4.1. Review Design

This manuscript was designed as a comprehensive narrative review aimed at providing a clinically oriented overview of current and emerging applications of artificial intelligence in cardio-oncology, with a focus on cancer therapy–related cardiotoxicity. Given the marked heterogeneity of study designs, populations, AI methodologies, data modalities, and outcome definitions in this rapidly evolving field, a formal systematic review or meta-analysis was not considered methodologically appropriate. Instead, a structured narrative approach was adopted to ensure broad coverage, clinical interpretability, and translational relevance.

### 4.2. Literature Search Strategy

A comprehensive literature search was conducted in the following electronic databases: PubMed/MEDLINE, Scopus, and Web of Science.

The search strategy incorporated relevant keywords and combinations thereof, including: cardio-oncology; cancer therapy–related cardiotoxicity; artificial intelligence; machine learning; deep learning; echocardiography; cardiac magnetic resonance; computed tomography; nuclear imaging; electrocardiography; radiomics; dosiomics; biomarkers; proteomics; genomics; extracellular vesicles.

Evidence was identified focusing on applications in electrocardiography, biomarkers, proteomics, extracellular vesicles, genomics, advanced imaging (echocardiography, cardiac magnetic resonance, computed tomography, and nuclear imaging), and radiotherapy dose modeling (dosiomics). Translational insights from animal models and in vitro systems were also included. In addition, the reference lists of relevant reviews and key original articles were manually screened to identify further pertinent studies.

### 4.3. Inclusion Criteria

Studies were considered eligible for inclusion if they met the following criteria:

1. Investigated applications of artificial intelligence, machine learning, or deep learning in the prediction, detection, monitoring, or prevention of cancer therapy–related cardiotoxicity or cardiovascular complications in oncology.
2. Included any study type, including clinical, translational, preclinical, in silico, or in vitro studies.
3. Addressed at least one of the following domains: cardiovascular imaging, electrocardiography, biomarkers, multi-omics, extracellular vesicles, or radiotherapy dose modeling.
4. Presented original research or methodologically relevant translational studies.
5. Used artificial intelligence for prediction, classification, segmentation, risk stratification, data integration, or decision support in a cardio-oncology–relevant context.
6. Were published in peer-reviewed journals.
7. No strict restrictions were applied regarding publication date in order to ensure a broad and comprehensive overview of the field.

### 4.4. Exclusion Criteria

Studies were excluded based on the following criteria:

1. Duplicate publications or overlapping reports from the same study population, in which case the most complete or most recent version was retained.
2. Articles whose title and abstract did not indicate a clear focus on artificial intelligence applications in cardio-oncology or cancer therapy–related cardiovascular toxicity.
3. Papers discussing artificial intelligence in oncology or cardiology without relevance to cardiotoxicity or cardiovascular complications of cancer therapy.
4. Studies unrelated to medical or biomedical applications.
5. Patents, book chapters, editorials, commentaries, opinion pieces, and non–peer-reviewed literature.

### 4.5. Data Extraction and Synthesis

From each selected study, we extracted information on the clinical context, data modality, artificial intelligence methodology, clinical task, validation strategy, reported performance metrics, and main limitations. The evidence was synthesized thematically and by modality, emphasizing clinical relevance, translational potential, and methodological trends rather than quantitative pooling of results.

### 4.6. Methodological Quality Considerations

Given the variable methodological quality and reporting standards in the artificial intelligence literature, studies were critically appraised with reference to TRIPOD-AI, PROBAST-AI, and CLAIM guidelines. These frameworks were used to contextualize common limitations, including small cohort sizes, risk of bias, lack of external validation, incomplete reporting, and limited assessment of calibration and clinical utility, rather than to formally exclude studies.

### 4.7. Rationale for Narrative Approach

This field is characterized by rapid technological evolution, heterogeneous methodologies, and a predominance of exploratory and proof-of-concept studies. Under these circumstances, a narrative synthesis was considered more appropriate than a systematic review or meta-analysis, allowing a broader, clinically meaningful, and integrative perspective while explicitly highlighting both opportunities and current limitations.

For clarity and synthesis, the main studies discussed in this review are summarized in modality-specific comparative tables covering echocardiography (Table 1), cardiac magnetic resonance (Table 2), computed tomography and radiomics/dosiomics (Table 3), nuclear medicine imaging (Table 4), electrocardiography (Table 5), and multimodal/translational approaches including biomarkers, genomics, proteomics, and extracellular vesicles (Table 6), highlighting for each domain the clinical task, AI methodology, study population, validation strategy, and reported performance.

## 5. AI in Cardiovascular Imaging

AI is being explored to enhance the precision, speed, and accuracy of LVEF and global longitudinal strain (GLS) assessment, assist in point-of-care image acquisition, and integrate imaging with clinical data to improve prediction and early detection of cardiac dysfunction. Additionally, AI applications in cardiovascular magnetic resonance (CMR), computed tomography (CT)—particularly coronary artery calcium (CAC) scoring—as well as nuclear medicine. Single Photon Emission Computed Tomography (SPECT) and Positron Emission Tomography (PET) imaging are emerging as promising tools for evaluating cardiac tumors and cardiovascular complications in both pediatric and adult cancer survivors [7].

### 5.1. Echocardiography

In cardio-oncology, AI has significant potential in echocardiography, supporting image classification, reconstruction, automated segmentation and quantification, and risk prediction through integration of clinical data [33].

Before cancer treatment: AI can play a critical role in the baseline cardiac assessment of patients prior to initiating potentially cardiotoxic therapies. Through automated analysis of echocardiographic parameters such as LVEF and GLS, AI can establish accurate and reproducible reference values. Moreover, AI algorithms can detect subtle structural or functional cardiac abnormalities that may not be readily apparent to the human eye, thereby enhancing diagnostic precision and improving the identification of patients at higher risk for future cardiotoxicity. Early identification of these risks can support more informed treatment planning and the implementation of preventive strategies [34]. During cancer treatment: throughout therapy, AI enables continuous or periodic cardiac monitoring by automating the measurement of LVEF and GLS, ensuring timely detection of even minimal changes in cardiac function. By integrating imaging findings with other clinical and biochemical data, AI can help predict cardiovascular outcomes and flag early signs of cardiotoxicity, allowing clinicians to intervene before irreversible damage occurs. Additionally, AI-assisted point-of-care imaging can standardize acquisition quality across institutions and operators, improving consistency in serial follow-up studies [34]. And finally, after cancer treatment, in the survivorship phase, AI continues to be valuable for long-term cardiac follow-up. Automated assessment tools can track functional recovery or progression of cardiac dysfunction over time and identify late-onset cardiotoxic effects that may emerge months or years after treatment completion. By integrating longitudinal imaging data with clinical and omic profiles, AI can help stratify survivors according to cardiovascular risk and guide personalized surveillance and prevention strategies [34].

By training convolutional neural networks (CNNs), Zhang was able to carry out image segmentation to pinpoint cardiac chambers and derive measurements of heart structure and function for constructing disease-classification algorithms. Zhang’s method is designed to unlock data mining and knowledge extraction from the massive repository of stored echocardiograms, promising substantial clinical value by integrating relatively inexpensive quantitative indicators into everyday medical workflows and enabling causal understanding that relies on consistent, long-term patient follow-up [35]. The study is considered foundational for applying automated interpretation to monitor patients over time, though its short analysis window and potential biases limit its strength. It also omits information on the gender distribution of participants, which could influence the findings.

After imaging registration and correct segmentation, automated measurements can be made [36]. Guidelines from the American Society of Echocardiography and the European Association of Cardiovascular Imaging recommend averaging five consecutive cardiac cycles to calculate LVEF. In clinical practice, however, the process is often time-consuming, so a single representative beat is typically used to estimate LVEF [37]. Therefore, the use of AI may have a definite impact on time-saving when calculating LVEF and other essential measurements. Another important challenge that AI has the potential to address is inter- and intra-observer variability, which affects all imaging modalities. For instance, studies have shown that LVEF measurements can differ between readers by as much as 10%. Notably, a 10% decline in ejection fraction is the same threshold used to define clinically significant cardiotoxicity, which may necessitate interruption of chemotherapy. This degree of variability poses a considerable challenge in cardio-oncology, as small reductions in LVEF are often attributed to measurement noise rather than true cardiac injury, potentially delaying detection of cardiotoxicity. Automation offers a solution to these issues by standardizing measurements and reducing variability [38,39]. Nonetheless, the role of the specialist remains indispensable for interpreting AI-generated results, applying clinical context, and ensuring the accuracy and reliability of automated assessments.

It is important to note that transthoracic echocardiography (TTE) has a more complex role than just appreciating GLS and LVEF, being an essential tool in cardio-oncology to assess ventricular, atrial, valvular, and pericardial structure and function in patients with current or past cancer [4,40].

In a large recent study, investigators assessed the use of AI for echocardiographic quantification. Using 877,983 measurements from 155,215 studies at Cedars-Sinai Medical Center (CSMC), they developed EchoNet-Measurements, an open-source DL model for automated annotation of 9 B-mode and 9 Doppler parameters. The model showed high agreement with sonographer measurements on internal CSMC and external Stanford Health Care (SHC) datasets (mean coverage probability 0.796–0.839; mean relative difference 0.096–0.120), with consistent performance across 2103 temporally distinct studies and various patient subgroups. These findings highlight AI’s potential to improve efficiency, accuracy, and workflow in cardiovascular imaging [41].

Another critical potential advantage and emerging role of AI in cardio-oncology lies in its potential to identify novel markers and predictive parameters of subclinical cardiotoxicity—well before traditional measures, such as LVEF and GLS, show any detectable changes [42,43].

A key application of AI in cardio-oncology imaging is automated LVEF measurement using AI-assisted point-of-care echocardiography, which can streamline workflow, enhance accuracy, and enable efficient real-time cardiac monitoring during oncology visits [33]. This approach enhances efficiency by eliminating the need for a cardiologist to be physically present at each visit, allowing reliable cardiac assessments to be performed seamlessly within oncology care settings. For example, recent studies have explored the use of AI-guided echocardiography performed by nurses with no prior ultrasound training. In one such study, each patient underwent paired limited echocardiograms—one acquired by a nurse guided by a DL algorithm and the other by an experienced sonographer without AI assistance. Five expert echocardiographers independently and blindly evaluated all studies. The results demonstrated that the DL algorithm enabled non-expert operators to obtain diagnostic-quality transthoracic echocardiograms suitable for assessing left and right ventricular size and function, as well as detecting nontrivial pericardial effusions [44].

One notable example of how AI may increase efficacy in cardio-oncology is the FAST-EFs multicenter study involving 255 patients, which demonstrated that automated LV measurements are feasible, rapid, and highly reproducible compared with traditional visual assessment and the manual Simpson’s biplane method. The average analysis time was only 8 ± 1 s per patient, with no inter- or intra-observer variability, highlighting the efficiency and reliability of AI-assisted echocardiographic analysis [33]. However, a limitation of the study lies in the relatively small cohort of patients.

GLS has been suggested as a more responsive indicator for the early identification of myocardial dysfunction before a measurable decline in LVEF occurs. However, some time ago, research by Farsalinos and colleagues highlighted considerable inconsistency across different echocardiography system manufacturers when assessing GLS [45]. To address this issue, Kwan and colleagues showed that an automated deep-learning strain (DLS) workflow can help harmonize measurements across different vendors, improving inter-vendor consistency irrespective of subjective image quality [46]. Moreover, a recent study demonstrated that, in a controlled setting, GLS measurements obtained from contemporary semi-automated clinical software are more consistent than they were a decade ago. Mid- and full-wall strain analyses were available in all but one software package. Endocardial as well as mid- and full-wall GLS measurements showed minimal inter-vendor variability, with an average maximum bias of only 0.6% strain units [47].

In a study by Kuwahara et al., a model utilizing a dedicated software application for echocardiographic analysis was used to evaluate left ventricular function in patients undergoing chemotherapy. The primary parameter analyzed was GLS, with additional assessment of LVEF, left ventricular dimensions, mass index, left atrial volume index, and diastolic function parameters such as septal and lateral e′ velocities. The AI-assisted model achieved an intraclass correlation coefficient (ICC) of 0.81 (95% CI: 0.64–0.90) between novice and experienced clinicians, compared to 0.62 (95% CI: 0.34–0.80) with conventional methods. These results highlight the ability of AI-derived tools to provide more consistent and reliable evaluations across clinicians with varying experience levels, reducing variability in the assessment of cardiac function and enhancing reproducibility of advanced echocardiographic measurements in the cardio-oncology setting [48].

In another study of 152 patients with HER2-positive breast cancer treated with anti-HER2 therapy and anthracyclines, AI-assisted analysis was used to obtain automated ejection fraction and GLS. These AI-derived values showed strong concordance with those obtained using standard software, with a median standard deviation of strain values of only 1.2% during serial echocardiographic monitoring, underscoring the accuracy and reliability of AI-based measurements [49].

Moreover, recently, Salte et al. evaluated another AI method for fully automated AI-based measurement of LV GLS in echocardiography. The AI successfully identified all three standard apical views and performed cardiac event timing in 89% of patients. It also automated segmentation, motion estimation, and GLS measurement across diverse cardiac pathologies and LV function ranges. GLS measured by AI was −12.0 ± 4.1% versus −13.5 ± 5.3% by the reference method, with a bias of −1.4 ± 0.3% (95% limits of agreement: 2.3 to −5.1), comparable to intervendor variability. The fully automated analysis eliminated measurement variability and was completed within 15 s [50]. AI-enabled, standardized GLS measurements across vendors could facilitate the detection of subtle, early signs of cancer therapy–related cardiotoxicity that may not yet produce measurable changes in LVEF.

A notable example of DL in echocardiography is EchoNet, a model trained on over 2.6 million echocardiogram images from 2850 patients. EchoNet demonstrated the ability to identify cardiac structures, assess function, and even predict systemic risk factors such as age and weight. The model accurately detected the presence of pacemaker leads (AUC = 0.89), left atrial enlargement (AUC = 0.86), and left ventricular hypertrophy (AUC = 0.75). It also provided reliable estimates of left ventricular end-systolic and diastolic volumes (R^2^ = 0.74 and 0.70, respectively) and ejection fraction (R^2^ = 0.50), while predicting age (R^2^ = 0.46), sex (AUC = 0.88), weight (R^2^ = 0.56), and height (R^2^ = 0.33). Interpretability analyses confirmed that EchoNet appropriately focused on key cardiac structures during explainable diagnostic tasks and highlighted novel regions of interest when predicting complex systemic phenotypes—suggesting that such AI models may uncover new, clinically relevant imaging biomarkers beyond human perception [42].

Another study presented EchoNet-Dynamic, a video-based AI model that analyzes full echocardiogram videos across multiple cardiac cycles. The model was trained on 10,030 annotated echocardiogram videos and demonstrated the ability to segment the left ventricle (Dice Similarity Coefficient 0.92), estimate ejection fraction (mean absolute error 4.1%), and classify heart failure with reduced ejection fraction (AUC 0.97). In an external dataset, performance remained strong, with a mean absolute error of 6.0% for ejection fraction and an AUC of 0.96 for heart failure classification. Prospective evaluation indicated variability comparable to or lower than that of human experts. By incorporating information across multiple cardiac cycles, EchoNet-Dynamic can assess subtle changes in ejection fraction with high reproducibility. The study also released the largest publicly available annotated echocardiogram video dataset to facilitate further research in AI-assisted echocardiography [51].

Cai et al. developed MMnet, a hybrid DL and ML model designed to automate the grading of diastolic function using echocardiographic data. The model analyzes key parameters, including mitral E and A wave velocities, septal and lateral e’ velocities, tricuspid regurgitation velocity, LVEF, and left atrial end-systolic volume, extracted from 2D grey-scale, pulse-wave, and tissue Doppler images. By integrating these features, MMnet delivers accurate and efficient diastolic function grading, demonstrating the potential of AI to enhance echocardiographic diagnostics with high precision and strong clinical applicability [52].

In a longitudinal prospective cohort study of 248 breast cancer patients receiving 240 mg/m^2^ of doxorubicin, supervised machine learning algorithms were applied to identify echocardiographic strain patterns most strongly associated with subsequent cardiotoxicity. Participants underwent 2D echocardiography at baseline, 4 months, and annually thereafter, with strain and strain rate analyses performed using *TomTec Cardiac Performance Analysis* software. The application of ML enabled the discovery of specific strain-based features predictive of cardiotoxicity, offering a promising approach for early detection of subclinical cardiac dysfunction in patients undergoing anthracycline therapy [53].

Chang et al. developed an AI-based predictive model using clinical, laboratory, and echocardiographic data from patients with newly diagnosed breast cancer, preparing for anthracycline therapy (2014–2018). The model incorporated 15 variables spanning clinical characteristics, chemotherapy regimens, and echocardiographic measurements, with the most influential predictors being trastuzumab use, hypertension, and cumulative anthracycline dose. The algorithm analyzed patterns in these data to identify patients at higher risk of CTRCD and heart failure with reduced ejection fraction. The study found that the model performed well in risk stratification, highlighting its potential to support early identification and management of patients susceptible to anthracycline-induced cardiotoxicity [54].

In a longitudinal retrospective study of 4309 cancer patients, echocardiographic data effectively predicted cardiac dysfunction, whereas laboratory data added little additional predictive value [55]. Echocardiographic data alone achieved an AUC of 0.85, compared with 0.74 for laboratory data alone. The combined model incorporating both data types performed best, with an AUC of 0.91 for diagnosing cardiac dysfunction [55]. The authors planned for the algorithm to be made available in an online risk stratification tool.

In a recent follow-up study, the research group applied ML to large-scale institutional electronic medical records to predict adverse cardiac outcomes in cancer survivors. They identified four clinically significant subgroups with distinct incidences of cardiac events and mortality, demonstrating that machine learning algorithms analyzing patient similarities over time can help identify survivors at increased risk of cardiac dysfunction [56].

Another promising application involves AI-driven re-analysis of stored imaging data within Picture Archiving and Communication Systems (PACS). Advances in AI image interpretation now allow automated review of previously acquired studies, improving diagnostic consistency and accuracy while reducing inter- and intra-observer variability [57].

Several vendors now provide AI-assisted tools for measuring LVEF. Evidence from a randomized clinical trial suggests that AI-based LVEF assessment may improve accuracy, efficiency, and reproducibility compared with conventional echocardiographic interpretation. Such tools have the potential to streamline clinical workflows, reduce inter-observer variability, and facilitate earlier detection of subclinical cardiac dysfunction [58].

**Table 1:** Key studies of AI applied to echocardiography.

Study	Task	AI Method	Cohort	Validation	Performance	Explainability	Main Contribution
Knackstedt et al. [33]	Automated LVEF & GLS	ML/DL	255 patients, multicenter	Internal	ICC > 0.9, time 8 ± 1 s	No	Fast, reproducible EF/GLS
Zhang et al. [35]	Full echo interpretation	CNN	~2850 patients	Internal	EF R^2^ ≈ 0.50, volume R^2^ ≈ 0.70	Partial	Foundational automated echo
Sahashi et al. [41]	18 echo parameters	DL	155,215 studies	External	Mean rel. diff 0.096–0.120	No	Large-scale automation
Salte et al. [50]	Automated GLS	DL	~200 patients	Internal	Bias −1.4 ± 0.3%, LoA −5.1 to 2.3	No	Eliminates variability
Kuwahara et al. [48]	GLS in chemo pts	DL	243 patients	Internal	ICC 0.81 vs. 0.62 manual	No	Improves reproducibility
Ouyang et al. [51]	Beat-to-beat EF	DL video	10,030 videos	External	MAE 4.1–6.0%, AUC 0.96	Partial	Video-based EF
Ghorbani et al. [42]	Structure + phenotype	DL	2.6 M images	Internal	Pacemaker AUC 0.89, LAE AUC 0.86	Yes	Shows biological attention
Cai et al. [52]	Diastolic grading	DL + ML	~1000 patients	Internal	Accuracy ~90% (reported)	No	Automated grading
Narang et al. [44]	AI-guided acquisition	DL	240 patients	External	Diagnostic quality in >90%	No	Enables non-experts
He et al. [58]	AI vs sonographers	DL	3769 studies	Prospective	Lower variability vs. humans	No	Workflow improvement
Cheng et al. [53]	Strain → CTRCD	ML	248 patients	Internal	AUC ~0.80	Partial	Early risk detection
Chang et al. [54]	CTRCD risk	ML	NA	Internal	AUC ~0.85	Partial	Multivariable risk
Zhou et al. [55]	CTRCD prediction	ML	4309 patients	Internal	AUC 0.91 (combined model)	No	Echo dominates prediction
Hou et al. [56]	Survivor phenotypes	ML	4632 patients	Internal	Distinct event curves	No	Risk stratification

AI = artificial intelligence; AUC = area under the curve; CNN = convolutional neural network; CTRCD = cancer therapy–related cardiac dysfunction; DL = deep learning; EF = ejection fraction; GLS = global longitudinal strain; HF = heart failure; ICC = intraclass correlation coefficient; LAE = left atrial enlargement; LoA = limits of agreement; LV = left ventricle; LVSD = left ventricular systolic dysfunction; MAE = mean absolute error; ML = machine learning; NA = not available; NPV = negative predictive value; R^2^ = coefficient of determination; ROC = receiver operating characteristic.

### 5.2. Magnetic Resonance Imaging

CMR is widely recognized as the gold standard for assessing ejection fraction and providing non-invasive tissue characterization, offering critical information to guide treatment decisions, particularly in patients receiving potentially cardiotoxic cancer therapy [59]. CMR can also give information/hints about the underlying mechanisms of cardiotoxicity.

A valuable study showed that DL-based fully automated analysis of left ventricular volumes and function is feasible, extremely fast and shows respectable performance without any manual corrections. Even with manual corrections—which are required for precise results in most patients—this approach remains time-efficient compared to manual analysis [60].

Fully automated cardiac localization and image plane acquisition are now commercially available, significantly reducing scan and analysis time while accurately detecting artifacts and enabling corrective actions or repeat acquisitions. AI-driven methods in parallel and real-time imaging, as well as compressed sensing, support faster image capture without loss of diagnostic accuracy. Furthermore, the use of AI in CMR tissue characterization—including radiomics and texture analysis—has enhanced the assessment of scar imaging, wall thickening, and inflammation [61].

A neural network has been developed to reconstruct cMRF T1 and T2 maps directly from undersampled spiral images in under 400 ms. The method is robust to varying cardiac rhythms, enabling rapid, real-time display of cMRF maps [62].

Edalati et al. evaluated two AI-based DL approaches for automated slice alignment (EasyScan) and cardiac shimming (AI shim) in cardiac MRI. The models were trained and validated on datasets from over 500 subjects. In subsequent prospective studies, AI-guided slice planning reduced operator dependence and shortened scan times by approximately 2 min (∼13% faster) compared to manual planning, while improving plane accuracy. AI shim enhanced B0 magnetic field homogeneity compared with conventional volume shimming. Overall, these AI tools demonstrated more efficient, standardized, and higher-quality cardiac MRI acquisition [63]. Despite not being built on dedicated oncologic patient populations, these studies underscore how their methodologies and insights could be meaningfully adapted to advance cardio-oncology practice.

The activity of some ICI displays cross-reactivity with cardiac proteins such as titin, which can determine myocarditis [64]. Of note, myocardial changes associated with ICI therapy are frequently initially subclinical and asymptomatic, which makes establishing a definitive diagnosis challenging. In addition, it may be difficult to ascertain whether early cardiotoxic changes reflect new toxicity or pre-existing myocardial damage [65]. Therefore, it is essential to use the most sensible methods in uncovering these subtle changes. CMR is most specific for tracking myocardial changes during or after myocarditis [66]. In some studies, artificial intelligence has been employed to detect early imaging changes suggestive of subclinical myocarditis. In one such study, early gadolinium enhancement (EGE) was assessed alongside left ventricular functional parameters using AI-based algorithms applied to CMR images from patients with acute myocarditis, highlighting a significant role for EGE, according to the Lake Louise criteria, in the evaluation of patients with a clinical suspicion of acute myocarditis [67].

Novel approaches, such as feature tracking, tagging and fast-strain-encoded CMR techniques are emerging means to assess myocardial strain using CMR [68].

Kar et al. investigated whether AI-derived GLS from left ventricular MRI could serve as an early, independent predictor of Cancer Therapy–Related Cardiac Dysfunction (CTRCD) in breast cancer patients receiving chemotherapy. Using DENSE MRI in 32 patients at baseline and 3- and 6-month follow-ups, two DeepLabV3+ fully convolutional networks automated LV segmentation and 3D strain computation. Cox proportional hazards models incorporating clinical and contractile factors demonstrated that GLS predicted CTRCD risk independently of LVEF. This AI-guided GLS approach may enable earlier identification of at-risk patients and guide cardioprotective strategies, though the study was limited by its single-center design and lack of external validation [69].

The StrainNet study investigated a convolutional neural network designed to perform myocardial strain analysis on cine CMR images from 161 healthy participants. The model demonstrated markedly improved accuracy in both global and segmental strain measurements compared with conventional post-processing techniques, highlighting its potential to enhance the precision and efficiency of CMR-based functional assessment [70].

Measurements of left atrial remodeling and vascular stiffness are increasingly accessible tools for assessing long-term cardiovascular risk following cancer therapy. For instance, in two studies of patients with hematologic malignancies treated with the tyrosine kinase inhibitor ibrutinib, abnormal left atrial strain and size on echocardiography, as well as elevated native T1/T2 values on cardiac MRI, were strongly predictive of future major adverse cardiac events and other complications [71,72]. Using AI to automate the calculation of these parameters could greatly accelerate the process and warrants evaluation in future studies.

In radiotherapy, cardiac substructure dose metrics are more predictive of late cardiac complications than whole-heart measures. Magnetic resonance-guided radiation therapy (MRgRT) allows visualization of substructures during daily localization, offering opportunities for improved cardiac sparing. One study extended the nnU-Net deep learning framework with self-distillation (nnU-Net.wSD) for substructure segmentation in MRgRT. Across 12 substructures, the model achieved a mean Dice similarity coefficient of 0.65 ± 0.25, outperforming a standard 3D U-Net (0.583 ± 0.28; *p* < 0.01), with better performance when leveraging fractionated data. Predicted contours generated dose-volume histograms closely matching clinical plans, with mean and maximum dose deviations of 0.32 ± 0.5 Gy and 1.42 ± 2.6 Gy, respectively. Volumes were largely consistent across institutions, with minor variability in coronary arteries. These results represent an important advance toward rapid and reliable cardiac substructure segmentation to enhance cardiac sparing in low-field MRgRT [73].

**Table 2:** Key studies of AI applied to cardiac magnetic resonance imaging.

Study	Task	AI Method	Cohort	Validation	Performance	Explainability	Main Contribution
Böttcher et al. [60]	LV volumes & EF	DL	50 patients	Internal	Dice ~0.94, small bias vs. manual	No	Fully automated LV
Hamilton et al. [62]	T1/T2 mapping	DL	Technical	Internal	Map error < 5%	No	Real-time mapping
Edalati et al. [63]	Planning & shimming	DL	>500 subjects	Prospective	~13% scan time reduction	No	Faster acquisition
Yuan et al. [67]	Myocarditis detection	ML/DL	21 patients	Internal	AUC ~0.90 (reported)	No	Detects EGE
Kar et al. [69]	GLS → CTRCD	DL	32 patients	Internal	HR significant; GLS predictive	No	Early CTRCD signal
Wang et al. [70]	Myocardial strain	DL	161 patients	Internal	Lower error vs. conventional	No	Better strain accuracy
Summerfield et al. [73]	MRgRT substructures	DL	18 patients	Internal	Dice 0.65 ± 0.25	No	Enables substructure sparing

AI = artificial intelligence; AUC = area under the curve; CMR = cardiac magnetic resonance; CTRCD = cancer therapy-related cardiac dysfunction; DL = deep learning; DENSE = displacement encoding with stimulated echoes; EF = ejection fraction; EGE = early gadolinium enhancement; GAN = generative adversarial network; GLS = global longitudinal strain; LoA = limits of agreement; LV = left ventricle; MAE = mean absolute error; ML = machine learning; MRgRT = magnetic resonance-guided radiotherapy; R^2^ = coefficient of determination.

### 5.3. Computed Tomography

Cardiac CT enables promising AI applications, such as automated CAC scoring on ECG-gated non-contrast chest CT, with multiple validated methods demonstrating high accuracy [74].

Detailed plaque characterization and quantification using CT offer valuable insights into various stages of coronary artery disease (CAD) [75]. The same technology, combined with AI applications, may play a pivotal role in uncovering cancer-related complications, like accelerated CAD.

Staging CT scans have typically been considered inadequate for assessing CAD risk, largely because they are not cardiac-gated. However, recent work demonstrates that AI applied to non-gated CT imaging can reliably estimate CAC scores. This development suggests that CAD detection and risk stratification may be incorporated into routine oncologic imaging without the need for additional scans, radiation exposure, or cost [76]. Shen et al. found that automated CAC derived from pre-treatment chest CT helped identify diffuse large B-cell lymphoma patients at higher risk for anthracycline-related cardiac dysfunction and MACE. However, the study was limited by a small sample size and inclusion of only Chinese patients, highlighting the need for broader validation [77].

On the other hand, deep learning-based calcium scoring methods classify individual voxels rather than candidate lesions. Because most voxels in CT images are background rather than CAC, Wolterink et al. proposed a two-stage approach using two convolutional neural networks: the first CNN identified candidate voxels in coronary CT angiography, and the second CNN further discriminated true CAC from other candidates [78].

An observational study of 315 non-contrast CT scans demonstrated that AI-based semi-automatic and automatic software produced Agatston, volume, and mass calcium scores, as well as the number of calcified lesions, with excellent correlation and agreement [79].

A recent study demonstrated that a DL–based algorithm for CAC scoring after chest radiotherapy could predict future acute coronary events (ACE). The study evaluated breast cancer patients who received adjuvant radiotherapy (*n* = 511) or did not (*n* = 600) between 2005 and 2013. CAC Agatston scores were analyzed using the AI algorithm, and the individual mean heart dose (MHD) was calculated, with no radiotherapy scored as 0 Gy. The primary endpoint was ACE following breast surgery. CAC scores were significant predictors of ACE, suggesting that AI-based CAC assessment on simulation CT could help identify high-risk patients, though further studies are needed to confirm these findings [80].

Interestingly, AI can generate a highly reliable and clinically useful cardiovascular disease risk profile from existing non-contrast chest CT scans in patients undergoing cancer treatment planning or follow-up. A DL model trained on 30,286 low-dose CT scans from the National Lung Cancer Trial successfully identified individuals at elevated risk of cardiovascular mortality (AUC 0.768), effectively transforming a lung cancer screening scan into a dual-purpose tool for cardiovascular risk assessment [81]. Larger studies evaluating the accuracy of AI-driven CAC and atherosclerotic disease assessment from already available CT scans in breast cancer patients are currently underway. The findings may also be applicable to individuals with other malignancies who undergo non-gated chest CT [7].

Coronary CTA radiomics identified invasive and radionuclide imaging markers of plaque vulnerability with good to excellent diagnostic accuracy, significantly outperforming conventional quantitative and qualitative high-risk plaque features. Coronary CTA radiomics may provide a more accurate tool to identify vulnerable plaques compared with conventional methods. Further larger population studies are warranted [82].

Gernaat et al. evaluated a CNN algorithm for the automated assessment of CAC and thoracic aorta calcification (TAC) in breast cancer patients undergoing CT scans for radiotherapy planning. The study reported high reliability of the CNN algorithm in quantifying both CAC and TAC, highlighting the potential of AI to facilitate cardiovascular risk assessment in oncology patients using imaging acquired for non-cardiac purposes [83].

Moreover, Gal et al. applied a DL algorithm for automatic quantification of CAC from CT scans in over 15,000 breast cancer patients scheduled for radiotherapy. The study demonstrated a strong correlation between the AI-derived CAC scores and cardiovascular risk, underscoring the potential of automated imaging analysis to enhance cardiovascular risk stratification in oncology populations [84].

Importantly, yet another emerging application of AI in cardio-oncology imaging lies in the optimisation of the detection of masses. AI can improve the evaluation of cardiac masses across detection, characterization, and monitoring. Because assessment of these masses relies on analyzing tumor size, shape, and textural patterns, AI’s ability to recognize complex—and sometimes imperceptible—image features is especially valuable. Through deep learning–based differentiation of healthy and cancerous tissue, AI can precisely measure tumor dimensions, define morphology, and accurately delineate mass margins, further expanding its role in cardio-oncology [85]. Additionally, algorithms can be incorporated, helping to determine the prognosis of the mass and to optimize its treatment [86]. Finally, AI can assist in monitoring treatment response by tracking changes in tumor size, texture, and the emergence of any new lesions [87].

Maffei et al. evaluated a radiomics-based AI classifier to assess the quality of automated segmentation of cardiac substructures for radiotherapy planning. Using 36 CT scans with 25 manually contoured substructures, radiomic features were extracted from both manual and automatic contours. A supervised-learning model was trained to distinguish correct from incorrect contours, achieving 82.6% accuracy and an AUC of 0.91. Key features showed strong correlation with standard quantitative metrics such as Dice index and Hausdorff distance. This approach demonstrates the potential for automated assessment of segmentation quality, which could support the expansion of autocontouring atlases and improve analysis of large radiotherapy datasets [88].

Another study developed and evaluated a DL approach for automatic segmentation of cardiac chambers, large arteries, and localization of the three main coronary arteries in CT scans used for radiation therapy planning. The method employed an ensemble of CNNs with two output branches, one for segmentation and one for coronary artery localization, trained on reference annotations and virtual noncontrast cardiac scans. Performance was assessed using Dice score (DSC) and average symmetric surface distance (ASSD), with 2D slice DSC ranging from 0.76 to 0.88 and ASSD from 0.17 to 0.27 cm, and 3D DSC from 0.87 to 0.93 and ASSD from 0.07 to 0.10 cm. Coronary artery localization achieved DSC values of 0.80 to 0.91. Predicted dosimetric parameters showed strong correlation with planned doses (R^2^ = 0.77–1.00 for chambers and large vessels; 0.76–0.95 for coronary arteries). The developed and evaluated method can automatically obtain accurate estimates of planned radiation dose and dosimetric parameters for the cardiac chambers, large arteries, and coronary arteries [89].

Lassen-Schmidt et al. evaluated an iterative training approach to improve AI-based segmentation of the heart and mediastinum using 132 thoracic CT scans annotated by 13 radiologists. In three training iterations, initial manual segmentations of 5–25 CTs were used to train a nnU-Net, with subsequent iterations incorporating AI-generated pre-segmentations corrected by humans. Model performance improved consistently across iterations, achieving Dice similarity coefficients of 0.91 for the heart and 0.95 for the mediastinum. The approach reduced human annotation time by 50% for the heart and 70% for the mediastinum, and even a model trained on just five datasets achieved DCS above 0.90. This iterative workflow demonstrates an efficient method for developing accurate AI segmentation models while progressively minimizing human effort, with future work focusing on optimizing initial dataset size and pre-processing strategies [90].

Jiad et al. implemented and evaluated an AI-based deformable image registration and organ segmentation method, termed AI dose mapping (AIDA), for estimating radiation dose to the esophagus and heart. The workflow required approximately 2 min per patient. Segmentations achieved mean Dice similarity coefficients of 0.80 ± 0.15 for the esophagus and 0.94 ± 0.05 for the heart, with Hausdorff distances at the 95th percentile of 3.9 ± 3.4 mm and 14.1 ± 8.3 mm, respectively. AIDA-derived heart doses were significantly lower than planned doses (*p* = 0.04), and larger dose deviations (≥1 Gy) occurred more frequently with AIDA (*N* = 26) than with manual dose accumulation (*N* = 6). The study demonstrates that rapid estimation of radiation dose to thoracic tissues using AIDA is feasible, with metrics and segmentations comparable to manual approaches, supporting its potential application in radiotherapy planning [91].

More recently, Borges et al. evaluated radiation dose distribution in auto-segmented cardiac substructures for left breast radiotherapy, emphasizing the importance of minimizing cardiac exposure as highlighted in the RTOG 1005 protocol. Anatomical structures were segmented using TotalSegmentator and Limbus AI, and the relationship between the cardiac area and other organs at risk was analyzed using log-linear regressions. The study found that dose-volume assessment protocols often overlook cardiac substructures, but automated tools can address these limitations. The authors correlated doses in the overall cardiac region with specific substructures, proposed planning limits, and suggested that statistical models could estimate doses for substructures lacking segmentation tools. Their findings also support the use of absolute dose-volume thresholds for future cause-effect evaluations [92].

**Table 3:** Key studies of AI applied to CT and radiotherapy planning.

Study	Task	AI Method	Cohort	Validation	Performance	Explainability	Main Contribution
Shen et al. [77]	CAC → CTRCD	DL	1468 patients	Multicenter	AUC ~0.75–0.80	No	Risk stratification
Gernaat et al. [83]	CAC/TAC scoring	CNN	~2300 patients	Internal	ICC > 0.90	No	Opportunistic screening
Gal et al. [84]	CAC → CV risk	CNN	15,915 patients	External	Strong HR gradients	No	Large-scale validation
Kim et al. [80]	CAC → ACE	DL	~1100 patients	Internal	CAC significant predictor	No	Post-RT risk
Chao et al. [81]	CV mortality	DL	30,286 patients	External	AUC 0.768	No	Dual-use CT
Kolossváry et al. [82]	Plaque vulnerability	Radiomics	25 patients	Internal	AUC 0.84–0.90	Partial	Radiomics > standard
van Velzen et al. [89]	Dose mapping	DL	18 patients	Internal	R^2^ 0.77–1.00	No	Accurate dosimetry
Maffei et al. [88]	Segmentation QC	ML	36 scans	Internal	AUC 0.91	Partial	Automated QC
Lassen-Schmidt et al. [90]	Heart segmentation	DL	132 scans	External	Dice 0.91–0.95	No	Robust segmentation
Jiang et al. [91]	Dose accumulation	DL	72 patients	Internal	Dice heart 0.94	No	Fast mapping

ACE = acute coronary event; AI = artificial intelligence; ASSD = average symmetric surface distance; AUC = area under the curve; CAC = coronary artery calcium; CAD = coronary artery disease; CNN = convolutional neural network; CT = computed tomography; CTA = computed tomography angiography; DL = deep learning; DSC = Dice similarity coefficient; ICC = intraclass correlation coefficient; ML = machine learning; MHD = mean heart dose; QC = quality control; RT = radiotherapy; TAC = thoracic aorta calcification.

### 5.4. Nuclear Medicine Imaging

Automatic CAC scoring methods have also been validated in other CT scan types that routinely visualize the heart, including attenuation correction images from PET-CT [93] and CT images used in radiotherapy treatment planning [94]. These findings suggest that cardiac PET may detect radiation-induced coronary artery damage. Consequently, applying AI to already available cardiac PET imaging to assess myocardial perfusion in cardio-oncology patients represents a promising emerging direction. ML-based risk prediction applied to PET scans outperformed logistic regression in identifying patients at high risk for myocardial ischemia and/or major adverse cardiovascular events, compared with the Systematic Coronary Risk Evaluation (SCORE) risk model derived from European Society of Cardiology guidelines [95].

It was proposed that AI could be used to monitor changes in the distribution of tagged 18F-FDG over time. If these changes can be characterized and linked to specific chemotherapy regimens and cardiovascular outcomes, targeted preventive strategies could be developed to mitigate such effects in future patients [96].

AI frameworks—including convolutional neural networks, U-Nets, and generative adversarial networks—have exhibited substantial promise in refining and augmenting PET and SPECT image quality. Most AI-driven enhancement techniques employ deep-learning models that accept a compromised or degraded image as input and reconstruct a cleaner, more diagnostically valuable output. In denoising applications, the input is marred by pronounced noise, whereas in deblurring or super-resolution tasks, the image is limited by diminished spatial resolution. Collectively, these approaches strive to restore sharper, more interpretable images and thereby strengthen the diagnostic utility and overall workflow efficiency within nuclear medicine [97].

Fluorodeoxyglucose F-18 (18F-FDG) PET imaging is commonly employed to detect cardiovascular complications related to ICI, which can activate cytotoxic T cells, aggravate underlying atherosclerotic processes, and elevate the likelihood of major cardiovascular events. AI-based approaches can facilitate the tracking of temporal shifts in 18F-FDG distribution in cancer patients, offering a more nuanced and sensitive means of identifying and monitoring ICI-associated cardiotoxicity [98].

**Table 4:** Key studies of AI applied to nuclear cardiac imaging.

Study	Task	AI Method	Cohort	Validation	Performance	Explainability	Main Contribution
Išgum et al. [93]	CAC from PET-CT	ML	133 patients	Internal	κ 0.85–0.89	No	CV risk from PET-CT
van Velzen et al. [89]	CAC from PET-CT	ML/DL	955 patients	External	High agreement vs. manual	No	Large-scale validation
Juarez-Orozco et al. [95]	Ischemia prediction	ML	1234 patients	Clinical	Internal	AUC ~0.80	Partial
Betancur et al. [96]	Obstructive CAD	DL	1638 patients	External	AUC ~0.80–0.85	No	Automated perfusion
Alves et al. [97]	Image quality	DL	13,844 records	Internal	SNR ↑, RMSE ↓	No	Better image quality
Calabretta et al. [98]	Vascular inflammation	ML/DL	12 patients	Internal	Higher sensitivity vs. visual	No	Tracks ICI toxicity

AI = artificial intelligence; AUC = area under the curve; CAD = coronary artery disease; CNN = convolutional neural network; DL = deep learning; FDG = fluorodeoxyglucose; ML = machine learning; MPI = myocardial perfusion imaging; PET = positron emission tomography; RMSE = root mean square error; SPECT = single-photon emission computed tomography; SNR = signal-to-noise ratio; ↑ = increase; ↓ = decrease.

## 6. AI Applied to Electrocardiography

Advances in computing power, machine learning techniques, and access to large-scale data may greatly enhance the clinical insights derived from the ECG while maintaining interpretability critical for medical decision-making in cardio-oncology [99]. Of note, ML algorithms demonstrate variability in sensitivity and specificity depending on the phenotype they target, with performance differing when identifying rarer, more severe cardiac conditions compared to more common, less severe ones [99].

In general, DL algorithms—especially those using CNN—demonstrated markedly better performance than rules-based methods and traditional ML approaches. These models show strong potential for analyzing resting ECG signals to detect structural heart disease, including left ventricular dysfunction. Such capabilities may support both population-level screening in asymptomatic individuals and earlier diagnosis in symptomatic patients, enhancing opportunities for timely intervention [99].

A landmark 2019 study from the Mayo Clinic trained a CNN using paired 12-lead ECG and echocardiogram data—LVEF as a measure of contractile function—from 44,959 patients to identify ventricular dysfunction defined as LVEF ≤ 35% using ECG data alone. When validated on an independent cohort of 52,870 patients, the model achieved an area under the curve of 0.93, with sensitivity, specificity, and accuracy of 86.3%, 85.7%, and 85.7%, respectively. Among patients without ventricular dysfunction, those with a positive AI screen had a fourfold increased risk (hazard ratio 4.1; 95% CI, 3.3–5.0) of developing future ventricular dysfunction versus those with a negative screen. This study highlights how AI applied to the ECG—an inexpensive and widely accessible test—can provide a powerful screening tool to identify asymptomatic left ventricular dysfunction [100,101,102,103].

In another study, AI-ECG-based predictions were combined with clinical risk factors, which further improved diagnostic accuracy (AUC 0.82), reinforcing the incremental value of ECG data in cardiovascular risk stratification. Among 14,613 participants in the Atherosclerosis Risk in Communities Study, 803 (5.5%) developed heart failure within 10 years. The deep-learning model using only 12-lead ECG data achieved an AUC of 0.756 (95% CI 0.717–0.795). Traditional risk calculators (ARIC, Framingham) achieved AUCs of 0.802 and 0.780, respectively. The best performance (AUC 0.818, 95% CI 0.778–0.859) was obtained when the ECG–AI output was combined with age, gender, race, body mass index (BMI), smoking, coronary disease, diabetes, blood pressure and heart rate in a light gradient boosting machine model—where the ECG-AI output emerged as the most important individual predictor. Together, these findings show that integrating AI-derived ECG signals with established clinical risk factors not only increases predictive accuracy but may outperform some traditional risk models [104].

Another research team evaluated whether AI-ECG could be integrated into a clinical decision-support tool for the early detection of cardiac dysfunction. In the study, 120 primary care teams from 45 clinics were assigned to either an AI intervention arm (181 clinicians) or control (usual care, 177 clinicians). ECGs from 22,641 adults without prior heart failure were analyzed. The primary outcome was new diagnosis of low ejection fraction (EF ≤ 50%) within 90 days. The intervention increased low EF diagnosis (2.1% vs. 1.6%; OR 1.32, *p* = 0.007) overall and among those with positive AI-ECGs (19.5% vs. 14.5%; OR 1.43, *p* = 0.01). Echocardiogram use was similar overall but higher in AI-positive patients in the intervention group (49.6% vs. 38.1%; *p* < 0.001). Results demonstrate that AI-enabled ECG screening improves early detection of low EF in routine primary care [105,106]. Similar models may be developed for cardio-oncology purposes.

Recent studies have evaluated the usefulness of AI-ECG for predicting incident atrial fibrillation in more than 1900 participants, using the Cohorts for Aging and Research in Genomic Epidemiology–AF (CHARGE-AF) score as a comparator. In this analysis, both the AI-ECG model output and the CHARGE-AF score independently predicted future AF. These findings suggest that AI-ECG may provide a convenient method for risk assessment using a single ECG, without the need for manual or automated extraction of clinical data [107].

AI can also extract subtle patterns from ECG images to detect conditions such as anemia, which may help in identifying higher-risk patient cohorts [108].

Although the studies described above were not conducted specifically in patients with a history of cancer, their findings suggest potential applicability to cancer survivors. To date, no studies have evaluated the use of AI-ECG to predict atrial fibrillation or other arrhythmias in cancer patients or survivors. Nevertheless, atrial fibrillation is increasingly recognized as a cardiovascular complication of antineoplastic therapy, occurring, for example, during ibrutinib or following chest radiotherapy [109].

In a recent retrospective study involving 459 patients, AI-ECG proved to be a strong screening tool for evaluating the risk of LVSD in individuals treated with anthracyclines or trastuzumab. Using a multivariable Cox regression analysis, the study found that a positive AI-ECG result independently predicted the development of LVD at 5 years [HR: 2.12 (95% CI, 0.66–0.72); *p* < 0.0001] [110]. Of note, it was proven that the AI-ECG algorithms have good results in detection of low ejection fraction, irrespective of ethnic and racial subgroups [111].

Over time, several studies have examined whether specific ECG patterns can predict cardiotoxicity following cancer treatment. One early study from 2001 evaluated children who developed cardiac dysfunction after receiving anthracycline therapy (198–737 mg/m^2^ cumulative doxorubicin equivalents). The investigators observed that a reduction in QRS duration occurred more frequently in children with cardiomyopathy than in both anthracycline-naïve cancer patients and healthy controls [112].

In 2019, a study of 589 children treated with anthracyclines found that a 0.6 mV reduction in the sum of absolute QRS amplitudes in the six limb leads (ΣQRS) and a 10 ms increase in QTc were associated with a 17% (HR 1.174; *p* = 0.003) and 10% (HR 1.098; *p* < 0.006) higher risk of developing cardiomyopathy—defined as LVEF < 50%, fractional shortening < 26%, or LV end-diastolic diameter z-score > 2.5—compared to children who did not develop cardiomyopathy. These ECG changes were more pronounced in patients receiving higher anthracycline doses [113]. Beyond flagging individuals at elevated risk for atrial fibrillation, deep learning applied to ECG data can also help identify patients susceptible to drug-induced QT prolongation. This capability may enable earlier recognition of patients who require closer monitoring or tailored therapy, particularly in settings where QT-prolonging oncologic or supportive medications are routinely used [114].

Bos et al. reported that AI-ECG surpasses the standard corrected QT interval in identifying LQTS, even among individuals with ECG-concealed forms of the syndrome, offering reliable insights into their underlying genotypic status [115].

A relatively recent study demonstrated that AI trained on electrocardiograms from 1217 childhood cancer survivors—mostly with lymphoma/leukemia and 77% having received anthracycline therapy (median dose 169 mg/m^2^, range 35–734 mg/m^2^)—can predict late-onset cardiomyopathy. In a test group of 244 high-risk individuals, the model achieved a sensitivity of 76%, specificity of 79%, and an AUC of 0.87 (95% CI 0.83–0.90), using the 2014 American Society for Echocardiography guidelines for cardiomyopathy definition. Results demonstrate that AI-enabled ECG screening improves early detection of low EF in routine primary care [116].

Jacobs et al. evaluated a CNN–based AI-ECG model to detect LVSD in breast cancer patients treated with anthracyclines, analyzing 1877 ECGs from 703 women with paired echocardiograms. The AI-ECG demonstrated strong diagnostic performance, achieving AUC values of 0.93 for LVEF < 50% and 0.94 for LVEF ≤ 35%, and showed excellent negative predictive value, making it particularly useful as an initial screening tool to determine which patients may require additional imaging. The model also tracked changes in cardiac function over time, suggesting usefulness for longitudinal monitoring. Importantly, the algorithm—originally developed in a general population—performed effectively in a cancer cohort, indicating good transportability [117].

Yagi et al. developed an AI-ECG model, “AI-CTRCD,” to predict chemotherapy-induced cardiac dysfunction in 1011 cancer patients receiving anthracyclines. About 8.7% developed cardiotoxicity, and a high AI-CTRCD score was associated with significantly increased risk (HR ~2.66), independent of clinical risk factors. The model improved predictive performance over conventional approaches, achieving a time-dependent AUC of 0.78 at 2 years, and consistently stratified risk across cancer types, sexes, baseline LVEF, and anthracycline doses, highlighting AI-ECG as a non-invasive tool for identifying patients at elevated cardiotoxicity risk [118].

In a recent study, Oikonomou et al. evaluated AI-ECG for detecting left ventricular dysfunction in patients with breast cancer or non-Hodgkin lymphoma treated with anthracyclines and/or trastuzumab. The AI-ECG model applied to baseline ECGs effectively stratified patients by risk: those with a positive AI-ECG had a 3.4-fold higher risk of overall CTRCD and a 4.9-fold higher risk of LVEF < 50% compared to negative screens. Longitudinal monitoring with sequential AI-ECG demonstrated dynamic changes in predicted risk, anticipating the onset of CTRCD and reflecting biologically meaningful structural cardiac changes. This was further supported by a significant correlation between AI-ECG–predicted echocardiographic GLS, a relationship that persisted even in patients with preserved LVEF [119].

**Table 5:** Key studies of AI applied to ECG for detection and prediction of cardiac dysfunction and cardiotoxicity.

Study	Task	AI Method	Cohort	Validation	Performance	Explainability	Main Contribution
Attia et al. 2019 [100]	Detect LVSD (EF ≤ 35%)	CNN	44,959 train + 52,870 validation	External	AUC 0.93; Sens 86.3%; Spec 85.7%	No	Landmark AI-ECG LVSD screening
Attia et al. 2019, 2021, 2022 [101,102,103]	LVSD detection (prospective & external)	CNN	Multinational cohorts	External, prospective	AUC ~0.90–0.93	No	Demonstrated transportability
Akbilgic et al. [104]	HF prediction	DL + GBM	14,613 ARIC participants	External	ECG alone AUC 0.756; Combined AUC 0.818	Partial	ECG-AI adds to risk models
Yao et al. [106]	Clinical deployment trial	CNN	22,641 primary care patients	Pragmatic RCT	OR 1.32 for new low EF detection	No	Improves real-world diagnosis
Christopoulos et al. [107]	Predict incident AF	DL	>1900 participants	Population-based	AI & CHARGE-AF both predictive	No	Single-ECG AF risk
Kwon et al. [108]	Detect anemia	DL	>50,000 ECGs	Multicenter	AUC ~0.90	No	Non-cardiac phenotyping
Rmilah et al. [110]	LVSD in chemo pts	CNN	459 cancer patients	Internal	HR 2.12 for future LVSD	No	First oncology-focused screening
Güntürkün et al. [116]	Late cardiomyopathy survivors	DL	1217 survivors	External	AUC 0.87; Sens 76%; Spec 79%	No	Predicts late toxicity
Jacobs et al. [117]	LVSD after anthracyclines	CNN	1877 ECGs/703 women	External	AUC 0.93 (EF < 50%), 0.94 (EF ≤ 35%)	No	High NPV screening tool
Yagi et al. [118]	CTRCD prediction	DL	1011 cancer patients	Internal	Time-dependent AUC 0.78; HR ~2.66	No	Predicts chemo cardiotoxicity
Oikonomou et al. [119]	CTRCD risk stratification	DL	1550 patients	Internal	HR 3.4 for CTRCD; HR 4.9 for EF < 50%	Partial	Tracks longitudinal risk
Noseworthy et al. [111]	Bias assessment	DL	44,959 patients	External	Stable performance across race	No	Addresses fairness
Bos et al. [115]	Long QT syndrome	DL	2059 patients	External	Outperforms QTc	Partial	Genotype-level detection
Prifti et al. [114]	Drug-induced QT risk	DL	1564 patients	External	AUC ~0.85–0.90	Partial	Predicts arrhythmia risk
Desai et al. [113]	Pediatric anthracycline risk	ECG features	589 children	Longitudinal	HR 1.17 per ΔQRS	No	Pre-AI ECG risk markers
Vaksmann et al. [112]	Pediatric cardiomyopathy	ECG	16 children	Observational	Significant QRS changes	No	Historical precursor

AF = atrial fibrillation; AI = artificial intelligence; AUC = area under the curve; BMI = body mass index; CI = confidence interval; CNN = convolutional neural network; CTRCD = cancer therapy–related cardiac dysfunction; DL = deep learning; ECG = electrocardiogram; EF = ejection fraction; GBM = gradient boosting machine; HF = heart failure; HR = hazard ratio; LV = left ventricle; LVEF = left ventricular ejection fraction; LVSD = left ventricular systolic dysfunction; ML = machine learning; NPV = negative predictive value; OR = odds ratio; QTc = corrected QT interval; ROC = receiver operating characteristic.

## 7. Multimodal and Translational Applications (Biomarkers, Genomics, Proteomics, and Extracellular Vesicle Analysis)

Conventional cardiovascular biomarkers, including cardiac troponins and brain natriuretic peptides (BNPs), have been studied for their ability to predict cancer therapeutics-related cardiac dysfunction [120]. In routine clinical practice, particularly among patients with low baseline cardiac risk, these assays are most valuable for their high negative predictive value, which ranges from 84% to 100% depending on the chosen threshold. However, they are very important for selecting the high-risk patients [121].

Initial research in patients with advanced or aggressive cancers treated with intensive cytotoxic chemotherapy found a link between increased troponin levels and later development of cardiotoxicity [122]. Randomized trials assessing combined BNP and troponin use in cancer therapy are lacking, and standardized thresholds for cardiotoxic troponin levels remain undefined, underscoring the need for assay harmonization [120].

In a prospective longitudinal study, serial measurements of high-sensitivity troponin I identified myocarditis in both symptomatic and asymptomatic patients receiving ICIs, highlighting its potential for early detection and prevention of adverse cardiovascular events [121]. Combining this information with AI might prove to be very useful for myocarditis prediction.

Biomarkers can be derived from various sources, including blood, urine, and tissue samples. Human-induced pluripotent stem cells (iPSCs), generated from patients’ somatic cells such as peripheral blood mononuclear cells, can be differentiated into relevant cardiovascular or immune cell types, offering a novel and versatile source of biomarkers [123].

Cardiovascular cells derived from iPSCs could offer a patient-specific, heart-focused model that preserves individual genetic information, permits repeated sampling, and eliminates the need for invasive procedures. They can be expanded in virtually unlimited numbers, facilitating personalized testing [123]. Nonetheless, iPSC generation and maintenance are resource-intensive and laborious, and the cells alone lack full physiological and environmental context. By integrating these models with high-throughput omics approaches—such as genomic, proteomic, and metabolomic profiling—and linking the data to detailed clinical phenotypes via bioinformatic analyses, researchers can uncover molecular or genetic signatures associated with heightened cardiotoxicity risk, providing a robust strategy for precision cardio-oncology [6].

While there is considerable promise in incorporating *genomics* into cardiotoxicity risk prediction, the evidence remains too limited and fragmented to support routine use in clinical cardio-oncology [124]. Transcriptional modification represents a difficult process that is pivotal to controlling gene expression and is challenging because of its dynamic and complex nature. In theory, DL models, which are good at working with large datasets and finding changes, offer an efficient solution for transcriptional modification analysis [125].

Recent studies have developed biologically relevant models to detect drug-induced toxicity through phenotypic screening. In one notable study, DL was applied to high-content image analysis of induced pluripotent stem cell–derived cardiomyocytes (iPSC-CMs) to rapidly identify patterns of cardiotoxicity. A library of 1280 bioactive compounds was screened, and a single-parameter DL score was used to flag compounds with potential cardiotoxic effects. Identified cardiotoxic agents included DNA intercalators, ion channel blockers, and inhibitors of EGFR, cyclin-dependent kinases, and multiple kinases [126].

Another study applied ML to develop a clinical and genetic risk prediction model for anthracycline-induced cardiotoxicity in childhood cancer survivors. The combined clinical-genetic model showed higher predictive accuracy and lower misclassification than a clinical-only model. More importantly, in vitro inhibition of gene-associated pathways, including PI3KR2 and ZNF827, protected cardiomyocytes from cardiotoxicity [127].

The use of iPSC-CMs in cardio-oncology research is expanding, including studies of electrophysiological effects of cancer therapies. Applying ML or DL algorithms to these electrophysiological changes could help predict—and potentially prevent—adverse cardiovascular events [128,129,130].

Several candidate single-nucleotide polymorphisms (SNPs) have been identified in mechanistic pathways implicated in anthracycline-induced cardiotoxicity, based on preclinical studies [131]. In addition, transcriptomic profiling using RNA sequencing, including single-cell RNA sequencing (scRNA-seq), has offered new insights into the biological mechanisms of cardiotoxicity. While bulk RNA sequencing provides average gene expression across a sample, scRNA-seq maps transcripts to individual cells, enabling single-cell resolution profiling in heterogeneous tissues [132]. When integrated with other single-cell multi-omics approaches—such as time-of-flight mass cytometry, T-cell receptor sequencing, and CITE-seq—scRNA-seq and spatial transcriptomics provide a powerful means to explore the cellular and transcriptomic landscape underlying cardiotoxicity [132]. Advances in high-throughput proteomics now allow the simultaneous quantification of hundreds to thousands of proteins from less than 100 μL of plasma. These methods have been explored in pilot studies of patients undergoing cardiotoxic cancer therapies, such as anthracyclines [133].

Im6Apred is an advanced model designed by Luo et al. in order to predict N6-methyladenosine (m6A) sites, which is an important RNA change that lies at the center of many biological processes [134]. The development of im6APred was based on the study of seven distinct classification methods, including four traditional algorithms and three DL techniques. This made it possible for im6APred to study the general methylation patterns on RNA bases and extend this knowledge to the entire m6A transcriptome across different tissues, with an increase in prediction accuracy [134].

Detecting Extracellular vesicles (EVs) is critical for the early diagnosis and prevention of common diseases, including cancer. Various techniques enable the identification and analysis of EV subtypes and their cargo, allowing differentiation between cancer-derived vesicles and those from healthy cells. EV levels are often elevated in the blood during chronic or acute inflammation associated with diverse diseases. Monitoring and characterizing EVs in circulation holds significant potential to transform their application as diagnostic and prognostic biomarkers [135].

By combining intelligent probabilistic systems, immunoaffinity separation, machine learning with nanosensors, and Raman scattering–based multifluidic devices, researchers can efficiently capture, precisely image, and trace EVs at the single-molecule level, greatly enhancing analytical sensitivity and resolution. These innovations provide powerful tools for disease diagnosis, therapeutic monitoring, and biomarker discovery, paving the way for advances in precision medicine and personalized therapy [136].

For example, Jin et al. developed a cancer diagnostic approach based on biomarker (circulating exosomes) detection in body fluids, creating a breast cancer liquid biopsy platform that integrates a fluorescence sensor array with a DL model called AggMapNet. By analyzing fluorescence spectral data, the system can accurately classify different cancer cells, offering a promising non-invasive strategy for breast cancer diagnosis [137].

On the other hand, Yuan et al. applied proteomic analysis combined with machine learning to develop a method for isolating tumor-derived extracellular vesicles from the blood of patients with non-small cell lung cancer (NSCLC). Their analysis identified a panel of seven proteins capable of reliably distinguishing healthy individuals from NSCLC patients, offering a valuable tool for early cancer detection [138].

In addition, Xie et al. introduced, for the first time, a rapid and visually detectable method called freeze–thaw-induced floating patterns of gold nanoparticles, which surpasses current technologies by improving the limit of EV detection by 100-fold. Remarkably, it enables multi-dimensional visualization of EVs through site-specific oligonucleotide incorporation, allowing accurate identification of EVs from different breast cancer subtypes using AI algorithms. This AI-enhanced micro-visualization approach provides a fast and precise point-of-care platform suitable for both basic research and clinical applications [139].

Koo et al. developed the ZAHV-AI system, which integrates the zeolite-amine and homobifunctional hydrazide-based EV isolation (ZAHVIS) platform with AI-driven analysis to improve colorectal cancer (CRC) diagnosis. The ZAHVIS platform allows rapid, simple, and cost-effective EV isolation, along with one-step extraction of EV-derived proteins and nucleic acids. Using plasma samples from 80 CRC patients and 20 healthy controls, they identified four EV-derived miRNA biomarkers (miR-23a-3p, miR-92a-3p, miR-125a-3p, and miR-150-5p) and combined these with carcinoembryonic antigen (CEA) levels in an AI-driven model. The optimal combination (miR-23a-3p, miR-92a-3p, miR-150-5p, and CEA) achieved an overall AUC of 0.9861, outperforming individual markers and conventional CEA tests. Impressively, the system achieved perfect detection for stages 0–1 (AUC: 1.0) and high accuracy for stage 2 (AUC: 0.9722) and early-stage CRC (AUC: 0.9861) using stage-specific combinations. The ZAHV-AI system thus represents a reliable, clinically relevant tool for CRC diagnosis, substantially enhancing early detection and monitoring [140].

**Table 6:** Key studies of AI applied to integrating biomarkers, genomics, proteomics, iPSC models, and extracellular vesicles for cardiotoxicity prediction and translational research.

Study	Domain	Task	AI Method	Cohort/Data	Validation	Performance	Clinical/Translational Relevance
Cardinale et al. [122]	Troponin	Predict cardiotoxicity	Statistical	High-dose chemo patients	Prospective	Troponin+ predicts LV dysfunction	Historical biomarker foundation
Demissei et al. [121]	Troponin/BNP	Myocarditis & dysfunction	Statistical	Breast cancer, ICI	Prospective	High NPV (84–100%)	Early detection
Grafton et al. [126]	iPSC-CM imaging	Drug cardiotoxicity screening	DL	1280 compounds	Internal	High accuracy DL score (qualitative)	High-throughput drug safety
Chaix et al. [127]	Clinical + genetics	Anthracycline cardiotoxicity	ML	Pediatric survivors	Internal	Better than clinical-only model	Precision risk stratification
Güntürkün et al. [116]	ECG + phenotype	Late cardiomyopathy	DL	1217 survivors	External	AUC 0.87	Long-term risk screening
Luo et al. [134]	Epitranscriptomics	m6A site prediction	Ensemble DL	Multitissue RNA	Internal	Higher accuracy vs. classical ML	RNA modification mapping
Salekin et al. [125]	Transcriptomics	Epitranscriptome prediction	GAN	Large RNA datasets	Internal	Improved representation learning	Transcriptional regulation
Huang et al. [132]	Single-cell omics	Mechanism discovery	ML/bioinformatics	scRNA-seq, multi-omics	Exploratory	Descriptive	Mechanistic understanding
Liu et al. [133]	Proteomics	Anthracycline toxicity	Statistical + ML	Patient plasma + mice	Internal	Hemopexin predictive	Biomarker discovery
Jin et al. [137]	EVs/Exosomes	Breast cancer detection	DL	Fluorescence spectra	Internal	High classification accuracy	Liquid biopsy
Yuan et al. [138]	EV proteomics	NSCLC detection	ML	Patient plasma	Internal	7-protein panel, high AUC	Cancer detection
Xie et al. [139]	EV imaging	Breast cancer typing	AI vision	Nanoparticle EV assay	Internal	100× sensitivity improvement	Point-of-care diagnostics
Koo et al. [140]	EV miRNA + CEA	CRC detection	AI ensemble	80 CRC + 20 controls	Internal	AUC 0.986; Stage 0–1 AUC 1.0	Near-clinical liquid biopsy
Perry et al. [128]	iPSC-CM	Risk stratification	ML concept	Lab models	Conceptual	N/A	Personalized cardio-oncology
Salem et al. [129]	iPSC-CM	Drug electrophysiology	Translational ML	Experimental	Internal	Mechanistic	QT/drug safety
Millard et al. [130]	iPSC-CM	Proarrhythmia screening	Statistical/ML	Multicenter	Reproducible	High concordance	Regulatory toxicology
Al-Otaibi et al. [131]	Genetics	SNP risk	Bioinformatics	Preclinical + clinical	Exploratory	N/A	Genetic susceptibility

AI = artificial intelligence; AUC = area under the curve; BNP = B-type natriuretic peptide; CI = confidence interval; CTRCD = cancer therapy–related cardiac dysfunction; DL = deep learning; EV = extracellular vesicle; GAN = generative adversarial network; iPSC = induced pluripotent stem cell; iPSC-CM = induced pluripotent stem cell–derived cardiomyocyte; ML = machine learning; m6A = N6-methyladenosine; N/A = not available; NPV = negative predictive value; RNA-seq = RNA sequencing; scRNA-seq = single-cell RNA sequencing; SNP = single nucleotide polymorphism.

## 8. Limitations and Challenges

A very important challenge and limitation is the need for high-quality, well-annotated data to train AI models. If the underlying datasets contain biases or inaccuracies, these flaws can be embedded into the algorithms, ultimately producing predictions that are unreliable [141].

A major challenge in the field is the substantial heterogeneity across existing studies. Study designs vary widely, with both prospective and retrospective approaches, and many lack a multidisciplinary framework. Differences in patient populations, cancer types, racial composition, cardiovascular risk profiles, definitions of treatment-related cardiotoxicity, biomarker assays, and imaging techniques further complicate comparisons. Additional limitations include small or selected cohorts, lack of external validation, restricted accessibility to imaging or AI tools, and variability across vendors and software platforms. This heterogeneity introduces potential selection and reporting biases, limits the generalizability of findings, and underscores the need for standardized, multicenter studies with larger, more diverse populations to develop broadly applicable clinical guidelines.

A key challenge in developing and implementing AI-based clinical decision support tools for ECG is infrastructural unpreparedness. Most AI-ECG models require access to raw digital time-voltage ECG data, which are often not available through electronic health records and remain unused in over 99% of institutions. Additionally, these raw data are frequently proprietary to vendors, further complicating access for research purposes. While these limitations are not unique to cancer survivors, studies in this population often face the additional challenge of small sample sizes [124]. Another challenge, in developing risk predictive scores, as stated in the Statement by AHA, lies in the fact that the pathophysiologic mechanisms underlying cardiovascular risk are highly individualized, reflecting a complex interplay between the specific malignancy, its treatment, and each patient’s variable susceptibility [124].

Moreover, the adoption of AI in healthcare introduces significant regulatory and ethical considerations, particularly regarding data privacy, patient safety, and the need for clear accountability in how algorithms are developed, validated, and used [142]. A key limitation remains the “black box” nature of many AI models, which obscures how predictions are generated and can hinder clinician trust and adoption.

In the United States, notable disparities persist in the health outcomes of racial and ethnic minority groups, largely driven by unequal access to healthcare and the broader influence of social and economic determinants. AI systems can unintentionally reinforce these inequities when structural biases within the data lead to unfair advantages or disadvantages—a phenomenon known as “algorithmic bias”. When minority and underrepresented populations are insufficiently represented in the clinical datasets used to train AI models, the resulting algorithms may struggle to produce reliable or equitable predictions for these groups [143].

It is important to recognize that publication bias may affect the current literature, as studies reporting positive results are more likely to be published than those with negative or inconclusive findings. This can lead to an overly optimistic perception of AI performance. Therefore, findings should be interpreted cautiously, and future research should emphasize transparent reporting of both successful and unsuccessful AI applications.

Despite the rapid expansion of artificial intelligence applications in cardio-oncology, the clinical adoption of these tools remains strongly influenced by issues of interpretability, explainability, and trust. Many of the most powerful models currently used in medical imaging, electrocardiography, and multimodal data integration rely on deep learning architectures that function largely as “black boxes,” meaning that the internal reasoning leading to a given prediction is not directly accessible to the clinician [144,145]. In cardio-oncology, this limitation is particularly important because AI outputs may influence high-stakes decisions, such as whether to interrupt, modify, or continue potentially life-saving cancer therapies, or whether to initiate cardioprotective treatment. In such contexts, clinicians must not only know what the model predicts, but also have a reasonable understanding of why a given patient is classified as high or low risk.

From a conceptual perspective, explainability methods can be broadly divided into two main categories. Intrinsic (or interpretable-by-design) models are constructed in a way that their decision process is inherently transparent, as is often the case with simpler machine learning models based on a limited number of clinically meaningful variables. In contrast, post hoc explainability methods attempt to provide explanations after a complex model has generated its output, for example, by highlighting image regions, signal segments, or input features that contributed most strongly to a given prediction. While such approaches can improve human understanding of model behavior, they do not fully eliminate the fundamental opacity of deep learning systems.

In cardiovascular imaging and ECG analysis, post hoc visualization techniques are increasingly used to illustrate which regions of an image or which portions of a signal drive a model’s prediction. When these highlighted areas correspond to clinically plausible structures, such as the myocardium, valves, or specific ECG segments, they may increase clinician confidence and help detect spurious correlations. However, these methods remain indirect and do not guarantee that the model’s reasoning is physiologically sound or robust across populations [144,145].

Another closely related issue is the limited assessment of uncertainty and failure modes in most current studies. Very few published models in cardio-oncology explicitly report prediction uncertainty, analyze cases of systematic failure, or define situations in which the algorithm should not be trusted. This is problematic, as overconfident but incorrect predictions may lead to inappropriate reassurance or unnecessary treatment modifications [146].

Importantly, most AI studies in cardio-oncology focus primarily on performance metrics such as AUC, Dice score, or mean absolute error, while providing little or no systematic evaluation of interpretability, explainability, robustness, or uncertainty. As a result, even models with apparently excellent performance may remain difficult to integrate into routine clinical workflows.

For AI tools to be safely and responsibly adopted in cardio-oncology, future studies will need to move beyond accuracy alone and place greater emphasis on transparency, clinical interpretability, uncertainty estimation, and failure-mode analysis. These elements are essential not only for regulatory approval and guideline integration, but also for building the trust of clinicians who ultimately remain responsible for patient care [145,146].

## 9. Conclusions and Future Directions

Artificial intelligence is accelerating the expansion of cardio-oncology into a new era that moves decisively beyond detection toward prediction and prevention of cancer therapy–related cardiotoxicity. By integrating data from imaging, ECG, biomarkers, multi-omics, and radiotherapy dose maps, AI models can uncover subtle patterns of cardiac injury long before clinical dysfunction emerges, offering a more comprehensive and dynamic view of patient risk. These innovations not only enhance diagnostic precision but also reduce variability, supporting more personalized and proactive cardiovascular care for patients undergoing cancer treatment. Future research should focus on harmonized multicenter datasets, rigorous external validation, and transparent reporting aligned with TRIPOD-AI, PROBAST-AI, and CLAIM. Equally important are efforts to address algorithmic bias and ensure that AI tools benefit diverse patient populations. As these advances converge, AI-enabled cardio-oncology has the potential to transform clinical practice—shifting from reacting to cardiotoxicity to anticipating and preventing it, ultimately improving both cardiovascular and oncologic outcomes for cancer survivors.

## Data Availability

No new data were created or analyzed in this study.

## Abbreviations

The following abbreviations are used in this manuscript:

AF	atrial fibrillation
AUC	area under the curve
BMI	body mass index
CAC	coronary artery calcium
CAD	coronary artery disease
CCTA	coronary computed tomography angiography
CMR/CMRI	cardiac magnetic resonance/cardiac magnetic resonance imaging
CNN	convolutional neural network
CT	computed tomography
CTRCD	cancer therapy–related cardiac dysfunction
CV	cardiovascular
D-BCC-sEVs	doxorubicin-treated breast cancer cell small extracellular vesicles
DL	deep learning
DENSE	Displacement Encoding with Stimulated Echoes
ECG	electrocardiogram
EGE	early gadolinium enhancement
EV	extracellular vesicle
FFRCT	fractional flow reserve computed from CT (FFR from CCTA)
GLS	global longitudinal strain
Gy	Gray (unit of radiation dose)
HFA	Heart Failure Association
HR	hazard ratio
HUD	handheld ultrasound device
ICI	immune checkpoint inhibitor
IC-OS	International Cardio-Oncology Society
iPSC	induced pluripotent stem cell
iPSC-CM(s)	induced pluripotent stem cell–derived cardiomyocyte(s)
LAD	left anterior descending (artery)/left descending artery
LVEF/EF	left ventricular ejection fraction/ejection fraction
LV/RV	left ventricle/right ventricle
LVSD	left ventricular systolic dysfunction
MACE	major adverse cardiovascular events
m6A	N6-methyladenosine (RNA modification)
MHD	mean heart dose
ML	machine learning
MRI	magnetic resonance imaging
MRgRT	magnetic resonance–guided radiation therapy
NAMs	New Approach Methodologies (non-animal methods referenced)
NT-proBNP	*N*-terminal pro–brain natriuretic peptide
OCT	optical coherence tomography
PACS	Picture Archiving and Communication System
PCI	percutaneous coronary intervention
PET	positron emission tomography
PMN	pre-metastatic niche(s)
PROBAST-AI	Prediction model Risk Of Bias ASsessment Tool-AI extension
QTc	corrected QT interval
RT	radiotherapy
sEV	small extracellular vesicle(s)
SPECT	single-photon emission computed tomography
TAPSE	tricuspid annular plane systolic excursion
TME	tumor microenvironment
Tn	troponin
TRIPOD-AI	Transparent Reporting of a multivariable prediction model for Individual Prognosis Or Diagnosis-AI extension
VCG	virtual control group
3Rs	Replace, Reduce, Refine (principles for animal research)
